# Contextualisation of the safeTALK™ Suicide Prevention Program: A Descriptive Qualitative Study

**DOI:** 10.1111/hex.70605

**Published:** 2026-02-16

**Authors:** Rita Pokharel Poudel, Diana Jefferies, Sheeja Perumbil Pathrose, Peter M. Gutierrez, Lucie M. Ramjan

**Affiliations:** ^1^ School of Nursing and Midwifery Western Sydney University Penrith New South Wales Australia; ^2^ Department of Psychiatric Nursing BP Koirala Institute of Health Sciences Dharan Koshi Nepal; ^3^ LivingWorks, Executive Vice President, Research & Development Englewood Colorado USA; ^4^ Department of Psychology Florida State University Tallahassee Florida USA; ^5^ School of Nursing University of Wollongong Wollongong New South Wales Australia

**Keywords:** adolescents, community‐based participatory research, suicide prevention

## Abstract

**Background:**

Suicide prevention programmes have effectively reduced suicidal behaviours, increased knowledge and fostered help‐seeking. However, suicide is a complex phenomenon, and its risk and protective factors differ across cultures.

**Objective:**

To contextualise the LivingWorks safeTALK™ suicide prevention programme for secondary school students in Nepal.

**Methods:**

A qualitative descriptive approach using focus groups informed by the Socio‐Ecological Model and guided by Community‐Based Participatory Research (CBPR) was used. The study was conducted in Nepal via Zoom™ from Australia, from September 15–30, 2024. Five focus groups were conducted with 18 participants, including adolescents, schoolteachers, parents, healthcare providers, and policymakers. Data were transcribed, translated to English and uploaded to NVivo v.14. A hybrid content analysis approach was used.

**Results:**

Data were presented across seven categories: 1. Factors contributing to suicide in Nepal, 2. The need for adolescent suicide prevention programmes in Nepal, 3. Stigma around suicide in Nepal, 4. Attitude towards adolescent suicide and the safeTALK™ suicide prevention programme, 5. Recommended modifications to the safeTALK™ programme 6. Readiness to implement an adolescent suicide prevention programme in Nepal, and 7. Challenges in implementing an adolescent suicide prevention programme in Nepal. Participants reported the need for adolescent suicide prevention in Nepal is high, and contributing factors to suicide are influenced by cultural practices and socioeconomic conditions. Further stigma around suicide prevents adolescents from seeking help. Some modifications to the existing programme were suggested by the participants. Some of the suggestions included translating the programme to Nepali and inclusion of Nepalese cultural practices. Minor modifications were made following approval from LivingWorks Australia.

**Conclusion:**

Suicide prevention is a major issue and requires further research to develop a culturally appropriate suicide prevention programme, especially among at‐risk adolescents in Nepal.

**Patient or Public Contribution:**

Adapted CBPR gave voice to the community, and led to dialogue and learning, and participatory decision‐making that were reflected in the contextualised safeTALK™ suicide prevention programme.

## 1 Background

The population suicide rate in Nepal was reported to be 23.56/100,000 in 2023 [1]. However, a school‐based survey in Nepal found that over 10% of adolescents aged 11 to 18 years had attempted suicide. The same study highlighted a lack of culturally appropriate prevention programmes [2]. Countries that have implemented school‐based suicide prevention programmes have effectively reduced suicidal behaviour [3, 4, 5, 6], increased knowledge about suicide, and fostered help‐seeking behaviour [7, 8, 9, 10, 11]. These programmes have instilled confidence in students to offer help to others who may be considering suicide [12, 13]. The safeTALK™ programme, developed by LivingWorks, is one of the prevention programmes that has been reported to be effective in increasing knowledge about suicide, a willingness to talk about suicide and help‐seeking intentions among school students in many countries, such as Canada, Australia, and the United Kingdom [7, 14, 15, 16, 17, 18].

While many school‐based interventions are prepared by experts, when the programmes are implemented in schools, there has been no evidence that stakeholders were involved as co‐researchers during the programme development. Furthermore, the programmes are not adapted for other cultures. As the theoretical models providing guidelines for suicide prevention research are derived from data obtained in Western cultures, many programmes are less likely to work in Low‐ and Middle‐Income Countries (LMICs) without contextualisation based on local cultures. This is because there are substantial differences in terms of gender roles and other sociocultural beliefs and practices from one culture to another [19]. To ensure that the prevention strategy is compatible in a different culture and context, the process of contextualisation can be employed to modify or adapt an existing evidence‐based programme to a different culture and context [20]. This is a crucial step as suicide is a complex phenomenon and its risk factors, protective factors, and preventive approaches differ across diverse cultures [2]. Lakshmi and Michale highlight a need to modify and adapt programmes implemented in High‐Income Countries before they are implemented in LMICs [19]. However, there are currently no contextualised suicide prevention programmes available to adolescents in LMICs [21]. Therefore, this study reports on the contextualisation of an existing suicide prevention programme— LivingWorks safeTALK™—for secondary school students in Nepal.

## 2 Aim

The aim of this study was to explore the socio‐cultural adaptations necessary to contextualise the safeTALK™ suicide prevention programme for use among secondary school students in Nepal.

## 3 Methods

### 3.1 Study Design

A qualitative descriptive approach was used to understand the needs and views of local people regarding adolescent suicide prevention in Nepal. This approach provided a comprehensive understanding of the needs of adolescents in Nepal, their risk factors for suicide, and the required approach for prevention. The qualitative descriptive design is typically selected when direct accounts of phenomena are required, and little is known about the topic. This is a flexible approach which provides a rich and comprehensive understanding of the phenomenon that stays close to the participants' own words [22, 23]. This study adhered to the Consolidated Criteria for Reporting Qualitative Research (COREQ) checklist [24] (Supporting File 1).

### 3.2 Theoretical Framework

**FIGURE 1:**
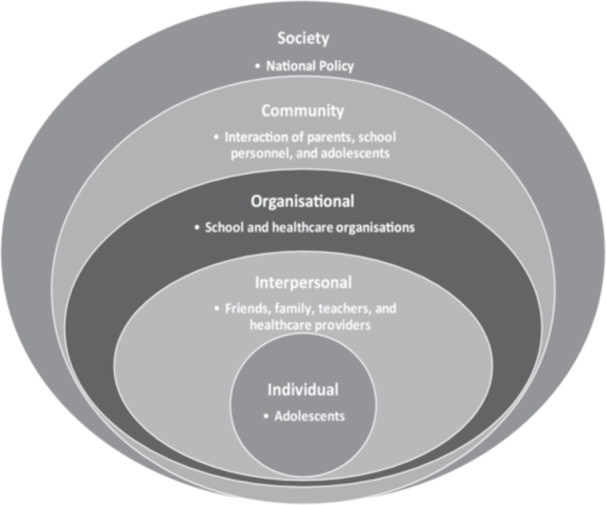
Socio‐ecological model (SEM).

Understanding that suicide is a complex, multilayered sociocultural phenomenon, this study was informed by the Socio‐Ecological Model [25] as the guiding framework (Figure 1). The core principles of the model are that multiple factors influence human behaviour and it requires a coordinated multilevel approach to modify such behaviours [25, 26, 27]. Suicide is a public health problem that requires a multilevel approach to prevent it. Through collaboration with the service provider LivingWorks Australia, adolescents, parents, teachers, school nurses, healthcare providers, and policymakers participated in this study to co‐develop a culturally appropriate version of the existing safeTALK™ suicide prevention programme.

### 3.3 Sociocultural Adaptation of the Programme

#### 3.3.1 The Programme

safeTALK™ is an educational programme (a half‐day workshop) designed to assist learners, aged 15 years or above, to recognise when another person is giving invitations, that is, inviting the person to explore their experience of suicidal thoughts. The person can then respond to the other person by applying basic *TALK* steps and connecting the other person to resources that can keep them safe. The name safe *TALK* is made up of two acronyms “safe” stands for s*uicide alertness for everyone* and “TALK” stands for *Tell Ask Listen and Keep Safe*. The programme includes presentations, videos, discussions, questions, and role‐plays to ensure that participants can provide safe and effective assistance to someone experiencing suicidal thoughts [28]. The programme has been shown to increase helpers' knowledge about suicide, belief that suicide is preventable, and willingness to help in several studies in Australia [7, 15, 17]. This study did not involve delivering the full programme. Instead, it focused on identifying the socio‐cultural adaptations needed to contextualise the programme for future use among secondary school students in Nepal.

### 3.4 Research Team

The research team consisted of a novice to expert qualitative researchers. The interviewer holds a Master of Science in Psychiatric Nursing degree and is a PhD candidate. She had prior experience of working as a Registered Psychiatric Nurse in Nepal, and undertook LivingWorks safeTALK™ [28], LivingWorks Applied Suicide Intervention Skills Training (ASIST) [29], and safeTALK™ training for trainers [30] prior to conducting the study. She also had some prior experience in research with young people and qualitative data collection. The PhD candidate was supported by more experienced qualitative researchers (DJ and LR) during the initial focus groups, data categorisation and writing up of the results (DJ, SPP and LR). All research team members (RP, DJ, SPP and LR) received safeTALK™ and ASIST training [28, 29].

The PhD candidate belongs to the same culture, and is a bilingual Nepali speaker; however, she did not have a relationship with the participants prior to the study. She was known to the participants as a Registered Psychiatric Nurse of Nepal and a PhD candidate at Western Sydney University, Australia.

### 3.5 Setting

This study was conducted in a public school in the Sunsari district of Koshi Province in Nepal. The school was conveniently selected for this study as per the study protocol [31]. This school is regarded as one of the region's most respected public schools and is the second largest in the province. It also attracts students from neighbouring districts and cities of the region, with more than 100 students studying in 1 year. There were 641 students studying in grade 11 at the time of this study. Nepal is a low‐income South Asian country and ranks 110th on the Legatum Prosperity Index [32]. The country is in the central Himalayan region, enjoys five seasons, and receives monsoons from June to September, with 2500 mm of rain each year. Nepal is a culturally diverse country with 50 different religious festivals celebrated with a fixed date marked in the Nepalese calendar. However, festivals are not confined to these dates; there are other festivals celebrated locally by ethnic groups. Public holidays are held on the most important days of those festivals, and they can be either local or national holidays. Dashain, Tihar, and Chhath Parva are the biggest festivals in Nepal, and at this time of the year (October to November), there is a long national holiday season, and most of the schools are closed during these festivals.

In this study, the researcher considered how to best plan travel to Nepal, considering the tourist season, weather, students' curricular activities, the school calendar, including the national board exam for school level, and festival closures in Nepal prior to data collection.

### 3.6 Sampling

Purposive, convenience, and snowball sampling techniques were used to recruit participants for this study. Students studying in grade 11 in a public school in Sunsari district, their parents, teachers, school nurses, mental health professionals, and representative health policymakers were the participants in this study. Grade 11 students were selected, as recommended by the school principal, to prevent disruptions to the academic schedules of students preparing for national‐level board examinations. The sample size was informed by Malterud's concept of information power, which suggests that the level of information power is influenced by five key factors: (1) study aim, (2) sample specificity, (3) use of established theory, (4) dialogue quality, and 5) analysis strategy [33]. This study's aim was narrow and focused on the phenomenon of interest, sample specificity was high, a theoretical model (SEM) informed the analysis, dialogue quality varied but overall focus group discussions were robust, and an exploratory cross‐case analysis was employed.

### 3.7 Recruitment

Participants were recruited across five different groups (adolescents, teachers, parents, healthcare providers, and policymakers).

To recruit the adolescents and teachers, the researcher visited the school (research site) and met with the school principal. The principal allocated one of the teachers to support the coordination of the focus group. Participant information sheets (PIS) and consent forms for parents, along with recruitment flyers, were distributed in the classroom. If the parents agreed to allow their child to participate and signed a consent form, a second process of consent was undertaken for the students themselves. The recruitment process was completed by the research support person. The research team also obtained consent from the teachers and the school nurse to be part of a second focus group.

A snowball technique was used to recruit parents of adolescents, and they were contacted via telephone and given further information at a face‐to‐face meeting. The researcher distributed the PIS and received consent from the parents after explaining the study.

Healthcare providers and policymakers were recruited through professional contacts via telephone, email, Facebook Messenger, and WhatsApp to inform them about the study. A PIS and consent form were sent to each participant, and focus groups were conducted in a group. However, one healthcare provider and one policymaker were interviewed one‐on‐one as they were unable to attend their focus group because of their busy schedules. Focus groups Open‐ended questions were informed by the adapted Community‐Based Participatory Research (CBPR) model developed by Belone et al. [34]. Consumers' participation in research improves sustainability by promoting ownership of the programmes by local people. In the context of the CBPR model, this study examined the socio‐economic, cultural, community, and readiness context. This model has been implemented and recommended by previous research in developing sustainable mental health programs [35, 36]. Furthermore, this model aligns with the concept of connectedness and the SEM model, which is a guiding framework for this study. To facilitate discussion, a booklet was prepared outlining the main elements of the safeTALK™ programme, and a guide for the questions and topics to be discussed and sent to the participants as pre‐reading 2 weeks before the focus group's date (Supporting File 2). Focus groups lasted from 17 to 98 min (about 1.5 h) depending on the number of participants in the group (mean duration of 49 min).

### 3.8 Data Collection

The researcher travelled to the research site to arrange logistics for the data collection, and to recruit participants from 29 August to 13 September 2024.

Focus groups over Zoom™ were conducted in the Nepali language from Australia after the researcher returned from Nepal. A total of five focus groups were conducted from 15 September to 30 September 2024. Two experienced researchers (LR and DJ) were present to guide the novice researcher during the initial three focus groups.

To commence the focus groups, the researcher introduced herself and other research team members to the participants, welcomed them to the session, opened with icebreaker questions, and explained the ground rules. Then the researcher provided an overview of the LivingWorks safeTALK™ programme, paused in between the presentation, discussed questions, and moved forward with the presentation.

The focus group with adolescents was conducted via Zoom™ in the computer classroom of the school. All students were in the same location on a single call. There was a research support person present in‐person throughout the session to ensure the safety of adolescents if they experienced any distress. The research support person is a registered nurse in Nepal, who is competent in providing basic mental health support. The adolescents were informed they could have the choice of any preferred adult in the room during the focus group. However, they were comfortable in the focus group with the research support person. None of the adolescents required any kind of support throughout and after the session. One schoolteacher was also present for technical support and coordinated the session with students in a computer classroom at the school.

### 3.9 Ethics

This research is registered in the Australia New Zealand Clinical Trial Registry (ACTRN 12624000634572). Ethical approval was obtained from the Human Research Committee of Western Sydney University (H15955), and Nepal Health Research Council (372_2024). The focus groups were audio recorded, and all recordings were de‐identified before analysis to ensure confidentiality.

### 3.10 Data Analysis

Focus groups were transcribed manually, translated into English and checked by two independent bilingual persons. After transcribing and translating the data into the English language, the two independent bilingual persons checked the data for accuracy, cultural appropriateness and conceptual equivalence. Then data transcription files were uploaded to NVivo v.15 for analysis. A hybrid content analysis approach was used to analyse the data [37]. The process of analysis included audio recordings being listened to several times by the researcher before transcribing the data to allow familiarisation. Researchers read and re‐read the data independently. Initial coding was done line by line, considering both the SEM and the adapted CBPR model, allowing the deductive creation of pre‐determined categories within existing frameworks and inductive categories generated from the data. Final categories were then reviewed by all team members (RP, DJ, SPP and LR) before writing the results.

The analysis of participants' perspectives on the recommended socio‐cultural adaptations, informed the contextualisation and refinement of the LivingWorks safeTALK™ programme for future use among secondary school students in Nepal. All recommended modifications identified in this study were discussed with the Chief Executive Officer of LivingWorks Australia for approval.

### 3.11 Research Rigour

Four‐dimension criteria were followed to ensure the rigour of this study [38]. The four dimensions were credibility, dependability, confirmability, and transferability.

To maintain credibility, the interviewer was familiar with the setting, the interviewer was trained by expert researchers, and more than two team members with prior experience in conducting focus groups were present during the focus groups. All the focus groups were conducted by the same person using a predetermined guide developed by the team of experts. Dependability was ensured by maintaining an audit trail of the study methods, and confirmability was enhanced through the use of a reflexive journal and peer review of analytical decisions by the research team. To achieve transferability, researchers have provided detailed descriptions of the study context, data collection and data analysis process, with illustrative quotes reporting the study findings.

### 3.12 Findings

**FIGURE 2:**
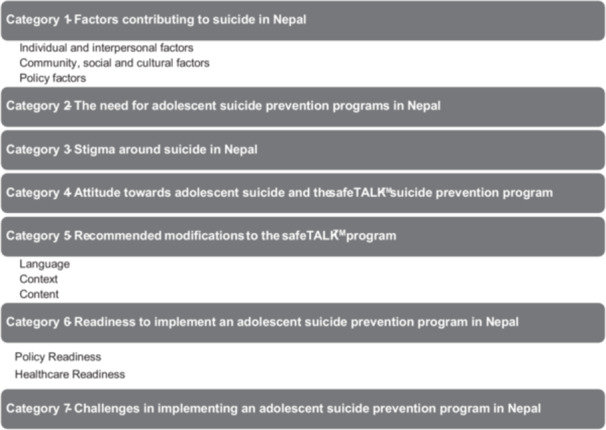
Categories and sub‐categories.

A total of 18 participants from the five groups participated in the focus groups (*n* = 7 adolescents, *n* = 2 teachers, *n* = 3 parents, *n* = 3 healthcare providers and *n* = 3 policymakers). The majority (55.55%) were female (Table 1). Data were analysed and presented as seven different categories and sub‐categories (Figure 2). Categories were based on the questions asked during focus groups (Supporting File 3). Exemplar quotes are presented in Table 2.

**TABLE 1:** Socio‐demographic characteristics of participants.

Characteristics	Adolescents (*n* = 7)	Parents (*n* = 3)	Teachers (*n* = 2)	Healthcare providers (*n* = 3)	Policymakers (*n* = 3)	Total (*n* = 18)
**Age** (Range)	16–17 years	—	—	—	—	—
**Gender**						
Female	5	—	1	2	2	10 (55.55%)
Male	2	3	1	1	1	8 (44.45%)
**Ethnicity**						
Janajati	5	—	—	1	—	6 (33.33%)
Bramhin/Chhetri	2	1	—	1	3	7 (38.89%)
Madheshi	—	2	2	—	—	4 (22.22%)
Dalit	—	—		1	—	1 (5.56%)
**Work Experience** (range)	NA	NA	8–12 years	2–5 years	5–18 years	NA

*Note:* NA: Work and experience data were not collected for the adolescents (students) and parents, as these variables were not relevant to the study objectives.

Age was collected only for the adolescents.

**TABLE 2:** Exemplar quotes related to the categories and sub‐categories.

Category	Sub‐category	Exemplar quotes
Factors contributing to suicide in Nepal	Individual and interpersonal factors	*“We do not have the tradition of talking about romantic relationships openly, romantic relationships are hidden from the family most of the time. There are a lot of pregnancies among teenage girls due to hidden sexual relations, after that they will break up the relationship and suicidal thoughts will arise.”* (P_9PM). *“It is very important in Nepal that suicidal thought is greatly affected due to poverty and unemployment”* (P_8PM) *“Parents do not support their children in the family. Parents will be provoked by the relatives when relatives make negative comments of children, due to lack of support from anywhere, there is more depression and suicide.”* (P_2AD) *“I think, if someone is a regular first ranker in the class, and failed suddenly one time, then there may be a situation of suicide.”* (P_2AD)
	Community, social and cultural factors	*“Early marriage and early pregnancy, discontinuing their education, so self‐sustainability cannot be achieved through employment, they cannot even make their own decisions, the husband and in‐laws also abuse them, they do not feel loved, and they choose suicide.” (*P_12HCP) *“When associated to Nepal's culture, the prevalence of superstition can increase suicide. For example, accusing someone of witchcraft, can also increase suicide among women.”* (P_6AD) *“The social stereotypical thought that males should not do the housework, and it is only the task of females in the Nepali family system, then the risk of suicide in this female is increased due to burden. The number of people attempting suicide is on the rise.”* (P_11HCP) *“There is a practice of caste untouchability, the lack of education may be responsible. Caste discrimination, such as not using water and other things touched by the lower caste makes people of the lower caste more upset, those painful feelings can lead to suicidal thoughts.”* (P_6AD) *“Chhaupadi and other forms of menstrual taboo also contributes to suicide.”* (P6_AD, P_12HCP, P_8PM). *In many communities of Nepal, females are no longer allowed to stay at home during menstruation. They need more love and care during these periods, instead they are discriminated by their family. So, this social cultural factor is also one of the reasons.”* (P_15SN) *“In my experience, it seems that people living in a nuclear family have more problems than those living in a joint family, living in a joint family may have been positive by sharing problems with each other or because the older people guide younger members in the family. In recent, years it seems to be difficult to stay in the joint family because of higher education and staying away from home for better job opportunities.”* (P_11HCP) *“As per my understanding, family size is being decreased, we are moving to nuclear family system, parents are at work, they spend less time with children. Maybe if there was a joint family, such problems would not have increased. Neighbours do not know what is going on in their neighbourhood, the family is being narrowed down, children are occupied with mobiles, they are ruined.”* (P_18P)
	Policy factors	*“Now the fertility rate is 71/1,000,000, there are a lot of pregnancies among teenage girls due to hidden sexual relations and inability to access family planning methods, which can lead to distress and lead to suicidal thoughts or mental health problems. Policy that supports the availability of family planning devices to everyone may reduce suicide among adolescent girls to some extent.”* (P_9PM)
The need for adolescent suicide prevention programmes in Nepal		*“Suicide continues to be a public health challenge in Nepal. If you look at this last year, about 2300 people lost their lives, the number of children and adolescents is significant. If we achieve sustainable development goals based on data, then we think that there is a big challenge. In the context of Nepal, there are about 24 deaths for every 100,000 people and our Goal is to reduce it to 4.7/100,000. Therefore, it is better to reduce child and adolescent suicide to 0. They are the future of the country, our future human resources. I said that if we could prevent suicide, it would be a great achievement for us. So, this type of programme is required”* (P_8PM) *“Now, suicide has been seen as a public health problem in Nepal as well. According to our latest information, Nepal has a suicide rate of 24/100,000 and the highest in the young and adolescent age group, so there is a need for a suicide prevention programme focusing on adolescence.”* (P_10PM) *“It is really needed for us because we have been challenged with more suicidal attempt cases and most of them are the adolescent age group and regarding prevention programmes, we focus on this during the suicide prevention day that is September 10 only and most of the programmes are being held during that time only, and no one is bothered by this issue at other times.”* (P_11HCP) *“The youth are the future of the country; if they start taking their lives, then only the elderly will live in the country and the country will not develop, instead the country will lag. As a result, there will be a big crisis in the country, so it is important to prevent suicide, this programme is needed.”* (P_2AD) *“Today, there is a high prevalence of suicidal thoughts, so this programme is especially important. It is important to do this programme as the problems of adolescent girls are understood by the people of the same age group*. (P_5AD) *“Such an awareness programme is particularly important because so far there is higher rate of female suicide as compared to men in Nepal, so there is a need for an awareness programme on suicide prevention.”* (P_13ST) *“It is necessary to raise awareness among everyone to help on time; this content should be included in social education of school curriculums so that it is quite easy for the students to understand such a situation and prevent suicide.”* (P_2AD) *“I believe that this education should be integrated into the curriculum of school‐level education.”* (P_17P)
Stigma around suicide in Nepal		*“People do not talk about suicidal feelings due to fear of exposure and being labelled in society, so it is difficult to assess their thoughts and understand suicidal feelings.”* (P_8PM, P_9PM) *“Mental problems are viewed by our society in a negative way, so it can increase the tendency of not expressing suicidal thoughts openly.”* (P_5AD) *“There is a negative attitude towards suicidal thoughts or mental problems, they label people as crazy for any type of problem, which can increase suicide.”* (P_2AD) *“When there is no‐one familiar or known person in the waiting areas of the clinic, only then they come to see the psychiatrist.”* (P_11HCP) *“Society does not take thoughts of suicide positively, teenagers cannot have an open conversation about problems in their minds, and it is a challenge to keep them safe.”* (P_13ST)
Attitude towards adolescent suicide and the safeTALK™ suicide prevention programme		*“In our society, families do not listen, do not believe that kids can commit suicide.”* (P_11HCP) *“If you talk about suicide, you are a coward, then you do not dare to speak openly about suicide, talking about mental problems and suicide is truly minimal in Nepal because the community does not accept it, family does not accept the person and feelings.”* (P_8PM) *“There is a concept that I am going on such a path, some people may cry, and that can lead them to more distress, their confidence may decrease. Therefore, I do not think that when asking about suicide, you must ask direct questions.”* (P_14ST) *“I think it would be better to ask in a twisted way than directly, if I am studying in 10th*–*11th grade, I do not understand anything and if you ask a straight question, then I feel that it will bring more suicidal thoughts in my mind. Don't you think that would increase suicide?”* (P_18P) *“Even though there is no policy and programmes in the country, you are doing this programme, I feel happy. Such a programme is necessary. We also learn something from this programme, and we can apply it here in our school as well*. (P_13ST) *“Suicide Prevention Programme is an incredibly positive programme. We have also learned a lot of things and we will also tell the children here, as you had shown the videos before, it is an incredibly good programme.”* (P_16P) *“The programme is a good workshop, I just saw on Google, it is good to say safeTALK. The word safeTALK itself is positive.”* (P_10PM)
Recommended modifications to the safeTALK™ programme	Language	*“It would have been more effective if it had been transformed into Complete Nepali.”* (P_2AD) *“It is better to show videos in Nepali, if the characters are also in Nepali, then it is better.”* (P_9PM) *“All the videos are suitable to be shown in Nepal, but if there would be Nepali subtitles, then it will be more effective.”* (P_11HCP) *“It is a good programme, and we can use it; it is more effective if the videos are Nepali subtitles.”* (P_12HCP) *“These videos need to be adapted to Nepali language and context.”* (P_10PM)
	Context	*“It would have been more effective if Nepali artists and Nepalese contextual scenarios could be in this video. If the people of Nepal were to be trained, then the effectiveness of the training would increase if they looked like Nepalese*.” (P_8PM) *“These videos need to be adapted to the Nepali language and context, those scenarios in the Western context, needs to be adapted to the Nepali context. Some things may be the same, but some things should be adjusted, for example, they [adolescents] went to cut grass or went to do fieldwork and talked about it [suicide]? There is a huge disparity in rural and urban settings of Nepal. It needs to be contextualised for rural scenarios as well.”* (P_10PM)
	Content	*“If you go to Madheshi or Muslim community, people are not open to talk to people of the opposite gender.”* (P_8PM) *“While saying active listening, some people may not feel comfortable with sustaining eye contact all the time during communication.”* (P_8PM) *“When you were telling the word INVITATION in the Tell section, I understood it as an invitation to suicide, so young people may understand it in the opposite way than your intended message.”* (P_18P) *“The word INVITATION needs to be explained in a contextual way*. (P_8PM, P_9PM, P_12 HCP)
Readiness to implement an adolescent suicide prevention programme in Nepal	Policy readiness	*“We are doing suicide prevention programmes in other modalities by mixing them with other programmes. We are investing a lot in mental health. We can implement it if there are strong policy recommendations. We can involve External Development Partners (EDPs) in supporting its implementation.”* (P_8PM) *“In our context, it is relevant, I see the scope to implement it in coordination with the school nurses, the Nepal Police have also worked on suicide prevention. I see the possibility of coordination with the local level government and the Ministry of Health. We can even train and mobilise Female Community Health Volunteers (FCHVs). It can also be done by talking to our EDPs.”* (P_10PM)
	Healthcare readiness	*“The biggest thing for me is that if the health worker does not know what to tell the teenagers about suicidal thoughts, then it would have been better if we could train health workers, then they could do it at the primary level if they know where to refer, suicide prevention can be improved. We must train health workers massively to implement suicide prevention strategies*. (P_9PM)
Challenges in implementing an adolescent suicide prevention programme in Nepal		*“There are no skilled health workers everywhere; it is difficult to ask or know the thoughts of suicide. If you talk about availability in Australia, then the availability of doctors may be remarkably high. We have problem with human resources and talking openly, so this year's theme on suicide prevention day (‘let us communicate openly’) applies a lot to us. Now in the middle of this video [in safeTALK™], they may be talking openly about community treatment, but if you look at the context of Nepal, people do not discuss at all, so it may have to be specified how to reduce stigma, and how to talk openly, before implementation.”* (P_8PM) *“Possibility of rolling out this programme throughout the country is a very tricky question. The Ministry of Health is positive, but there is also a lack of financial resources and other resources, this is a challenge. Even if EDPs start the programme, eventually it will be integrated into the government system, so we need to plan before implementing any programme. Strong policy recommendation is required.”* (P_10PM)

*Note:* EDP = International non‐governmental organisations that provide funding to health programmes. These organisations include the World Health Organisation and UNICEF

Abbreviations: AD, adolescent; HCP, healthcare provider; P, parent; PMP, policymakers; ST, school teacher; SN, school nurse.

### 3.13 Categories

**Category 1 Factors contributing to suicide in Nepal**

Some participants expressed that there are many sociocultural factors and cultural practices in Nepal which can lead to an increased rate of suicide.

**Individual and interpersonal factors:** Participants discussed the following factors that could lead to suicide: Poverty and unemployment; romantic relationships, which are not accepted by their families; different types of violence faced by individuals; hormonal and bodily changes among adolescents; and academic pressures.

**Community, social and cultural factors:** There are prevailing superstitious beliefs among the community based around the hierarchal caste systems and gender bias at home, including child marriage and a preference for male children in some families. These problems could be exacerbated by dowry traditions and menstruation taboos. A few participants also identified excessive mobile phone use and changes to the traditional family structure as factors that could lead to experiences of suicidal thoughts.

**Policy factors**: Some policy factors regarding sexual and reproductive health contribute to stress and can be a risk factor for suicide. For example, there are challenges for young people obtaining contraceptives, such as condoms, oral contraceptive pills or injections and intrauterine devices, because they may be afraid that they will be judged by staff in the health centres. One policymaker explained that adolescents are commonly engaged in romantic relationships, which are hidden due to social stigma and are not accepted by families. If they become pregnant outside of marriage, society will not accept them, and for this reason, adolescents may attempt suicide due to shame. If policies could be changed to allow access to contraceptives, through adolescent‐friendly health services, regardless of their marital status, suicide could be prevented to some extent.

**Category 2 The need for adolescent suicide prevention programmes in Nepal**

All participants expressed that there is a need for a structured programme for adolescent suicide prevention, as many participants reported that suicide among adolescents is on the increase. Adolescent girls are attempting suicide at a higher rate, and if a preventive approach can be implemented, adolescents will be more aware about what to do and how to access appropriate help for suicidal thoughts early. This could potentially prevent many suicides in the future.

**Category 3 Stigma around suicide in Nepal**

Participants described how stigma surrounding mental health and suicide manifests in Nepalese society and how this stigma and shame are a barrier to timely help‐seeking and effective prevention.

**Category 4 Attitude towards adolescent suicide and the safeTALK™ suicide prevention programme**

The majority of participants discussed general community attitudes and perceptions towards suicide as not being positive. They stated that many people in the community say suicide is the act of a cowardly person who is incapable of doing anything productive with their life. They also explained their own and the community's perspectives on asking direct questions about suicidal ideation. A few participants expressed hesitation in asking someone direct questions about suicidal ideation, and one participant believed asking direct questions could increase the risk of suicide. Despite this, all participants had a positive attitude towards the safeTALK™ suicide prevention programme.

**Category 5 Recommended modifications to the safeTALK™ programme**

Most participants suggested modifications to three areas of the existing safeTALK™ programme before implementing it in Nepal‐Language, Context and Content. The required modifications were discussed with LivingWorks Australia, and the final approved modifications are presented in Supporting File 4. For example, Nepali subtitles were added to the videos and cartoon animations were created based on participants' suggestions.

**Language:** Most participants expressed that the current videos would be more effective in the Nepali language, and a few participants suggested that at least Nepali subtitles would be better than not having any Nepali language in the videos. Nepali subtitles were added with the help of an expert to the main videos, which conveyed the key messages.

**Context:** Some of the participants suggested producing videos that depict Nepali characters/people and settings in Nepal that show the sociocultural context of Nepal and would enhance the effective delivery of the programme. Two short animations were developed based on the cultural practices of Nepal (Supporting File 5).

**Content:** Although none of the participants suggested reducing or removing any of the existing content, they suggested adding some content related to the sociocultural risk factors for suicide in Nepal. For example, discussion of cultural practices specific to the local community, like menstruation taboos and hierarchical caste systems, were included in appropriate sections of the safeTALK™ programme and presented to LivingWorks Australia as a proposed and approved modification either to the content or during delivery. A few important minor modifications to the content were made during delivery, such as explaining the word **“** ***Invitations*** **”** during translation into the Nepali language. This was important because a few participants perceived the word as an invitation for suicide rather than for prevention, and participants felt it may be interpreted by adolescents in this way. A few participants also suggested that one should be careful in maintaining eye contact in conversations because it can be considered disrespectful and could be a barrier to effective communication in some cultures. One participant mentioned that the trainer's gender could influence the effectiveness of the training in certain communities. For example, if the suicide helper is male and is asking questions to a female, the participant may not be open to the helper. So, this factor was important to discuss during the delivery of the training (Supporting File 4).

**Category 6 Readiness to implement an adolescent suicide prevention programme in Nepal**

All participants stated that there was a need for a suicide prevention programme in Nepal, demonstrating the readiness of people from both the individual and community levels. Some participants expressed the need for strong policy recommendations to implement an adolescent suicide prevention programme.

**Policy readiness:** Participants discussed how the government of Nepal has set a target to reduce the suicide rate, but currently, there are no structured adolescent suicide prevention programmes in Nepal. All policymakers reported that an adolescent suicide prevention programme is required in Nepal. A strong policy recommendation from researchers could provide positive attitudes among all policymakers and related government agencies for developing national‐level guidelines and implementation of a suicide prevention programme.

**Healthcare readiness**: A few participants reported that training is required for healthcare providers and that healthcare infrastructure must improve to implement any innovative programmes. However, if there is strong evidence, recommendations can be incorporated into existing programmes.

**Category 7 Challenges in implementing an adolescent suicide prevention programme in Nepal**

All policymakers highlighted gaps in collaboration among different government sectors and agencies, emphasising the need for coordinated efforts from health, education, financial and social sectors to effectively prevent suicide. They also reported that the sustainability of the programme was another challenge due to poor intersectoral collaboration, financial constraints, human resources, and limited infrastructure. A few participants also identified individual beliefs and stigma as challenges to overcome in the implementation of any suicide prevention programme.

## 4 Discussion

This study aimed to contextualise the existing safeTALK™ programme for use among secondary school students in Nepal. Sociocultural adaptations necessary to reflect the local context prior to implementation of the programme were identified in relation to three areas: language, context and content. In the contextualisation of a programme, suicidal issues such as risk factors and attitudes can vary across different sociocultural contexts. Cultural factors need to be considered in adolescent suicide [39, 40]. For example, Eastern Asian adolescents may attempt suicide due to shame in not meeting expectations of family or others, and acculturative stress is associated with high suicides among Latin American adolescents when they are living outside their home countries [39]. Additionally, cultural considerations may affect help‐seeking [41]. Therefore, some modifications seem important to consider before implementing a suicide prevention programme, such as safeTALK™, in a new setting to make it culturally appropriate for Nepal.

Suicide is a public health issue, and it requires a multi‐sectoral approach to its prevention. Therefore, this study used the Socio Ecological Model (SEM) as a guiding framework [25]. Guided by the SEM, this study involved stakeholders across multiple levels to contextualise the programme. Adolescents were intentionally positioned as the core stakeholders at the individual level through to policy level officers. This multi‐layered approach provided a rich understanding of diverse viewpoints.

The Lancet reported that there are multiple examples of the successes and challenges of using a public health model in preventing suicide globally [42]. Our study also reported different social and cultural perspectives, particularly in the Nepalese and South Asian context. For instance, non‐mainstream topics related to Nepali context in our study including the hierarchal caste system, untouchability, dowry tradition, and Chhaupadi or other forms of menstrual taboo (which all contribute to suicide in Nepal) can be learning opportunities for people with a limited understanding of the cultural nuances of Nepal and other South Asian countries and can also can be beneficial to future researchers or programme developers in South Asia to understand the importance of contextualisation.

For many people in South Asia, particularly in Nepal and India, the caste system plays a significant role in social exclusion [43]. The system consists of four main categories: “Brahmins,” priests; “Kshatriyas,” warriors; “Vaishyas,” merchants; and “Sudras,” servants. Below these groups are the Dalits, often referred to as the “untouchables.” Caste‐based discrimination remains a highly sensitive and politically charged issue in Asia. Nepal has attempted to combat caste discrimination by enacting laws that declare caste‐based discrimination a crime. However, discrimination persists in Nepali society, especially in rural areas, and includes behaviours such as refusing to enter Dalits' houses or allowing them to enter your house. Also, sharing food and water and refusing to touch people who are Dalits are common [43]. People who face daily discrimination can also experience mental stress, sometimes leading to suicide. These factors must be taken into account when adapting or tailoring a programme for use in another country to ensure contextual relevance and effectiveness.

Another significant factor contributing to suicide among South Asian girls and women is the dowry dispute [44], which was identified in the focus groups as a possible reason for girls and women to consider suicide. Participants in our study highlighted this issue to be discussed in the safeTALK™ prevention programme. Provision of a dowry is a common cultural practice in some communities of South Asian countries, where the groom's family expects money and valuable items from the bride's side, and this expectation brings the bride's family under extreme pressure. If expectations are unmet, different types of family violence against the woman may continue for many years after the marriage [44]. This can be a significant contributor to poor mental health and a higher rate of suicide among females in the South Asian context [44]. Therefore, gender‐based prevention strategies are recommended in Nepal.

A typical cultural practice observed in Western Nepal is Chhaupadi or menstrual exclusion [45]. However, different forms of menstruation taboo exist among Hindu communities throughout Nepal [45, 46]. Menstrual exclusion, such as ‘*Chhaupadi*’, is an extreme form of ‘untouchability’ in far‐western Nepal. Women and girls who are menstruating are forbidden from touching other people and objects, and are required to live away from the community, typically in cattle sheds, during menstruation. Despite being a crime under Nepali law, this tradition continues due to community‐based values, social beliefs and religious practices. Similar practices are common across many countries of South Asia, and they have multidimensional negative effects on the lives of girls and women and affects their mental health [45, 46]. This practice not only prevents them from participating in normal daily activities but also increases their risk of robbery and sexual assault during this period of menstrual exclusion. Therefore, suicide prevention programmes developed for such communities should also consider these issues.

Additionally, other cultural practices and superstitions need to be considered in the design and delivery of suicide prevention programmes for Nepalese society. Participants cited factors such as community preference to a male child and pressure on women to give birth to males. There are also superstitious beliefs among the community, including witchcraft practices. An accusation of witchcraft is one of the serious forms of violence in Nepal, especially with females [47]. Many experience serious physical injury and die due to physical abuse received by the people in the community. Such types of practices subject the victims and their families to profound distress, and they are often at high risk of suicide. Therefore, minor modifications were made to the existing safeTALK™ programme, so that typical Nepalese practices could be discussed during programme delivery and during group activities. However, further contextualisation may be required for the Nepalese context to address the diverse needs of the population.

In addition to cultural adaptation, language and contextual issues were raised by the participants. Language and nonverbal communication are also linked to culture. Language provides a strong sense of self‐identity, and of being connected to the community. Presenting suicide prevention programmes in the local language may promote connectiveness for young people to the programme. This should increase the acceptability and effectiveness of suicide prevention strategies [48]. To address language issues in the existing programme, the research team added Nepali subtitles to the English videos and planned for the trainer to translate and deliver all the training in the Nepali language. Furthermore, to address the contextual issues, we prepared two animations based on the cultural and contextual practices in Nepal. In addition, we included an explanation of the meaning of the word INVITATION used in the training package so that it delivered the intended message of the programme, as participants were concerned that adolescents might interpret the word in a way that is opposite to the intended meaning. During translation, the literal translation may not always deliver the intended message. We also incorporated an explanation of how to adjust the context of the programme to consider specific gender issues when asking questions in certain communities in Nepal. In some communities of Nepal, if the suicide helper belongs to the same gender of the person experiencing the problem, they can be more open to communication and feel more comfortable with the helper, leading to positive outcomes.

One participant also expressed that asking direct suicide questions can increase suicide; however, a previous study did not report any iatrogenic effects of direct questions [49]. This is an example of the prevalent myths around suicide in the community. The LivingWorks safeTALK™ programme has provided guidelines about asking direct questions when an invitation suggests someone is reaching out for support because they are experiencing suicidal thoughts.

Other adjustments included accommodating cultural differences, such as minimising eye contact during active listening to avoid feelings of being threatened in certain communities. In Nepal, maintaining too much eye contact is considered rude, while this is generally considered an active listening technique in Western cultures [50]. In addition, we replaced the existing list of numbers in the KeepSafe™ connections to local numbers for Nepal.

While participants expressed a clear and evident need for an adolescent suicide prevention programme in Nepal, there are a variety of challenges to overcome. The first challenge is working in a country where there are no policies and definite guidelines for suicide prevention. This makes it challenging to gain the trust of the community and service consumers to implement or test a new programme. National policies can be associated with accountability, and accountability is associated with public trust [51]. Other factors include stigma, or a negative attitude towards prevention programmes due to inadequate awareness and the prevalence of myths around suicide. Stigma is also associated with decreased help‐seeking [52]. Another major challenge is financial constraints in resource‐poor settings, especially when there is negligible government expenditure on health, and where the emphasis is on a curative rather than a preventive approach. Other challenges are the disparity between awareness levels of people from various parts of the country, huge economic variability, poverty and geographical difficulties to reach certain population groups. Despite various challenges, there are many opportunities for researchers and policymakers to work in this space. While previous studies implemented and evaluated the effectiveness of this programme, it was not contextualised to the Nepalese context [7, 15, 17, 21]. Although further work is needed before full implementation, piloting the contextualised version in the Nepalese community provides an important first step.

## 5 Strengths and Limitations of the Study

This study is the first to explore the needs of a community and to contextualise the safeTALK™ programme for use among secondary school students in Nepal and the first documented study to contextualise an existing programme for adolescent suicide prevention in LMICs. By involving multiple stakeholders, including adolescents, the robustness of this study and acceptance within the local community was enhanced. This study gives voice to the community, and led to dialogue and learning, and participatory decision‐making, reflected in the recommendations for the contextualised version of the safeTALK™ programme. However, limitations include that the study was based on the urban area of eastern Nepal, and therefore, it may not address the cultural complexities of suicide affecting populations in other areas and indigenous communities. Furthermore, a larger and more diverse participant pool, combined with extended time for deeper focus group interaction, could have enhanced the analytic breadth of the findings. Another limitation was not being able to modify certain parts of the safeTALK™ programme for future implementation because of resources constraints and copyright issues. For example, while participants recommended changes to the people featured and the contextual scenarios depicted in the existing videos, these modifications were not possible. As an alternative, in addition to the existing videos, we produced two short animations for future implementation (Supporting File 5).

## 6 Implications for Policy and Practice

This study has highlighted the need for adolescent suicide prevention policy guidelines and programmes in Nepal and other LMICs. More importantly, this study has highlighted the importance of contextualisation of programmes before implementation, to promote equity and relevance at the policy level, and the potential for improved engagement, uptake and sustainability within the local culture. To date, there are no national‐level adolescent suicide prevention strategies or policies documented in Nepal. Implementation of this programme in schools, as part of a broader suicide prevention strategy, teaches people to be alert and supports the connection of people experiencing suicidal thoughts to helping resources. It is an important part of a community's efforts to reduce suicide and should contribute to a national policy for adolescent suicide prevention in Nepal.

## 7 Recommendations for Further Research

Using a rigorous programme development methodology, the contextualised version should first be tested against the standard version and the learning outcomes compared. If, as hypothesised, it demonstrates better outcomes, implementation would be warranted, followed by evaluation of programme effectiveness. Although significant work is needed before recommending full implementation, piloting the contextualised version presents an opportunity to generate baseline evidence in Nepal.

## 8 Conclusion

This study is the first to gather stakeholder perspectives to contextualise an existing structured suicide prevention programme for adolescents in Nepal. While all factors in the existing LivingWorks safeTALK™ programme remained the same, minor additions were made based on this study's findings prior to implementing and pilot testing the programme in a public school in Eastern Nepal. Suicide prevention is a major issue and requires further research to develop a culturally appropriate suicide prevention programme, especially among at‐risk adolescents in Nepal. This early evidence provides a critical foundation for subsequent, larger‐scale qualitative and mixed methods studies that can further refine, challenge, or extend the themes identified in this exploratory study.

## Author Contributions

**Rita Pokharel Poudel:** conceptualisation, methodology, data collection, data analysis, writing – original draft. **Diana Jefferies:** conceptualisation, methodology, focus groups, validation, writing – review and editing. **Sheeja Perumbil Pathrose:** conceptualisation, methodology, data analysis, writing – review and editing. **Peter M. Gutierrez:** writing – review and editing. **Lucie M. Ramjan:** conceptualisation, methodology, focus groups, validation, data analysis, writing – review and editing, supervision. All authors have read and agreed to the published version of the manuscript.

## Funding

The authors received no specific funding for this work.

## Ethics Statement

This study was approved by the Western Sydney University Human Research Ethics Committee (H15955) and Nepal Health Research Council (372_2024).

## Conflicts of Interest

Peter M. Gutierrez is an Executive at LivingWorks, but did not participate in the design, implementation, data analysis, or interpretation of the study. Their only involvement was in providing critical revisions to the final manuscript. The other authors declare no conflicts of interest

## Supporting information

Supplementary_file_1_COREQ_Checklist.

Supplementary_file_2_Booklet.

Supplementary_file_3_Focus_group_questions.

Supplementary_file_4_Approved_modifications.

Supplementary_file_5_Animation_videos.

## Data Availability

Data are available from the corresponding author on reasonable request because the data cannot be made publicly available due to the sensitive nature of the data, confidentiality and privacy concerns for participants.

## References

[1] G. Bhandari , L. Nath , P. R. Bhatt , R. Mishra , and A. Bhatt , “Exploring Trends: Five‐Year Analysis of Suicide Rates in Nepal,” Mental Illness 2024, no. 1 (2024): 5396303, 10.1155/2024/5396303.

[2] A. R. Pandey , B. Bista , R. R. Dhungana , K. K. Aryal , B. Chalise , and M. Dhimal , “Factors Associated With Suicidal Ideation and Suicidal Attempts Among Adolescent Students in Nepal: Findings From Global School‐Based Students Health Survey,” PLoS One 14, no. 4 (2019): e0210383, 10.1371/journal.pone.0210383. 31002715 PMC6474648

[3] R. H. Aseltine, Jr. , A. James , E. A. Schilling , and J. Glanovsky , “Evaluating the SOS Suicide Prevention Program: A Replication and Extension,” BMC Public Health 7 (2007): 161, https://ezproxy.uws.edu.au/login?url=http://ovidsp.ovid.com/ovidweb.cgi?T=JS&CSC=Y&NEWS=N&PAGE=fulltext&D=med6&AN=17640366. 17640366 10.1186/1471-2458-7-161 PMC1941734

[4] E. A. Schilling , R. H. Aseltine , and A. James , “The SOS Suicide Prevention Program: Further Evidence of Efficacy and Effectiveness,” Prevention Science 17, no. 2 (2016): 157–166, 10.1007/s11121-015-0594-3. 26314868

[5] E. A. Schilling , M. Lawless , L. Buchanan , and R. H. Aseltine, Jr. , “Signs of Suicide’ Shows Promise as a Middle School Suicide Prevention Program,” Suicide and Life‐Threatening Behavior 44, no. 6 (2014): 653–667, 10.1111/sltb.12097. 24796660

[6] D. Wasserman , C. W. Hoven , C. Wasserman , et al., “School‐Based Suicide Prevention Programmes: The SEYLE Cluster‐Randomised, Controlled Trial,” Lancet 385, no. 9977 (2015): 1536–1544, 10.1016/S0140-6736(14)61213-7. 25579833

[7] E. Bailey , M. J. Spittal , J. Pirkis , M. Gould , and J. Robinson , “Universal Suicide Prevention in Young People: An Evaluation of the safeTALK Program in Australian High Schools,” Crisis 38, no. 5 (2017): 300–308, 10.1027/0227-5910/a000465. 29098895

[8] A. L. Calear , M. Banfield , P. J. Batterham , et al., “Silence Is Deadly: A Cluster‐Randomised Controlled Trial of a Mental Health Help‐Seeking Intervention for Young Men,” BMC Public Health 17, no. 1 (2017): 834, https://ezproxy.uws.edu.au/login?url=http://ovidsp.ovid.com/ovidweb.cgi?T=JS&CSC=Y&NEWS=N&PAGE=fulltext&D=med14&AN=29061168. 29061168 10.1186/s12889-017-4845-z PMC5653993

[9] A. L. Calear , S. M. McCallum , H. Christensen , et al., “The Sources of Strength Australia Project: A Cluster Randomised Controlled Trial of a Peer‐Connectedness School‐Based Program to Promote Help‐Seeking in Adolescents,” Journal of Affective Disorders 299 (2022): 435–443, 10.1016/j.jad.2021.12.043. 34952104

[10] S. Freedenthal , “Adolescent Help‐Seeking and the Yellow Ribbon Suicide Prevention Program: An Evaluation,” Suicide & Life‐Threatening Behavior 40, no. 6 (2010): 628–639, https://ezproxy.uws.edu.au/login?url=http://ovidsp.ovid.com/ovidweb.cgi?T=JS&CSC=Y&NEWS=N&PAGE=fulltext&D=med8&AN=21198332. 21198332 10.1521/suli.2010.40.6.628

[11] P. A. Wyman , C. H. Brown , M. LoMurray , et al., “An Outcome Evaluation of the Sources of Strength Suicide Prevention Program Delivered by Adolescent Peer Leaders in High Schools,” American Journal of Public Health 100, no. 9 (2010): 1653–1661, https://ezproxy.uws.edu.au/login?url=http://ovidsp.ovid.com/ovidweb.cgi?T=JS&CSC=Y&NEWS=N&PAGE=fulltext&D=med8&AN=20634440. 20634440 10.2105/AJPH.2009.190025 PMC2920978

[12] L. M. Hart , P. Cropper , A. J. Morgan , C. M. Kelly , and A. F. Jorm , “Teen Mental Health First Aid as a School‐Based Intervention for Improving Peer Support of Adolescents at Risk of Suicide: Outcomes From a Cluster Randomised Crossover Trial,” Australian and New Zealand Journal of Psychiatry 54, no. 4 (2020): 382–392, 10.1177/0004867419885450. 31707787

[13] L. M. Hart , A. J. Morgan , A. Rossetto , et al., “Teen Mental Health First Aid: 12‐month Outcomes From a Cluster Crossover Randomized Controlled Trial Evaluation of a Universal Program to Help Adolescents Better Support Peers With a Mental Health Problem,” BMC Public Health 22, no. 1 (2022): 1159, https://ezproxy.uws.edu.au/login?url=http://ovidsp.ovid.com/ovidweb.cgi?T=JS&CSC=Y&NEWS=N&PAGE=fulltext&D=med22&AN=35681130. 35681130 10.1186/s12889-022-13554-6 PMC9185965

[14] B. Wilson and E. Neufeld , “SafeTALK Suicide Training: An Evaluation of Attitudes and Actions Among Medical Students,” Res Medica 24, no. 1 (2017): 4–16, 10.2218/resmedica.v24i1.1538.

[15] G. Holmes , A. Clacy , D. F. Hermens , and J. Lagopoulos , “Evaluating the Longitudinal Efficacy of SafeTALK Suicide Prevention Gatekeeper Training in a General Community Sample,” Suicide and Life‐Threatening Behavior 51, no. 5 (2021): 844–853, 10.1111/sltb.12741. 33594707

[16] S. Kerr , C. Martin , and M. Fleming , “Preventing Suicide; Nurse Education and the Occluded Issue of Gender,” Nurse education in practice 32 (2018): 58–63, 10.1016/j.nepr.2018.07.004. 30031273

[17] I. Kinchin , A. Russell , D. Petrie , A. Mifsud , L. Manning , and C. M. Doran , “Program Evaluation and Decision Analytic Modelling of Universal Suicide Prevention Training (safeTALK) in Secondary Schools,” Applied Health Economics and Health Policy 18, no. 2 (2020): 311–324, https://ezproxy.uws.edu.au/login?url=http://ovidsp.ovid.com/ovidweb.cgi?T=JS&CSC=Y&NEWS=N&PAGE=fulltext&D=med17&AN=31410773. 31410773 10.1007/s40258-019-00505-3

[18] R. J. Mellanby , N. P. H. Hudson , R. Allister , et al., “Evaluation of Suicide Awareness Programmes Delivered to Veterinary Undergraduates and Academic Staff,” Veterinary Record 167, no. 19 (2010): 730–734, 10.1136/vr.c5427. 21257507

[19] V. M. P. Lakshmi , “Suicide Prevention in Low and Middle‐Income Countries,” in The International Handbook of Suicide Prevention, eds. a J. P. Rory and C. O'Connor (John Wiley & Sons, 2016). 2nd ed. Incorporated, http://ebookcentral.proquest.com/lib/uwsau/detail.action?docID=4690025.

[20] M. J. Opozda , J. Bonson , J. Vigona , et al., “Navigating the Cultural Adaptation of a US‐Based Online Mental Health and Social Support Program for Use With Young Aboriginal and Torres Strait Islander Males in the Northern Territory, Australia: Processes, Outcomes, and Lessons,” International Journal for Equity in Health 23, no. 1 (2024): 165–115, 10.1186/s12939-024-02253-w. 39169369 PMC11337567

[21] R. Pokharel Poudel , S. Perumbil Pathrose , D. Jefferies , and L. M. Ramjan , “Effectiveness of Suicide Prevention Programmes Among Adolescents and Sociocultural Adaptation of Programmes: A Systematic Review,” International Journal of Mental Health Nursing 34, no. 2 (2025): e70038, 10.1111/inm.70038. 40207732 PMC11984072

[22] M. Sandelowski , “Whatever Happened to Qualitative Description?,” Research in Nursing & Health 23, no. 4 (2000): 334–340, 10.1002/1098-240x(200008)23:4<334::aid-nur9>3.0.co;2-g. 10940958

[23] P. Villamin , V. Lopez , D. K. Thapa , and M. Cleary , “A Worked Example of Qualitative Descriptive Design: A Step‐by‐Step Guide for Novice and Early Career Researchers,” Journal of Advanced Nursing 81, no. 8 (2025): 5181–5195, 10.1111/jan.16481. 39382252 PMC12271654

[24] A. Tong , P. Sainsbury , and J. Craig , “Consolidated Criteria for Reporting Qualitative Research (COREQ): A 32‐item Checklist for Interviews and Focus Groups,” International Journal for Quality in Health Care 19, no. 6 (2007): 349–357, 10.1093/intqhc/mzm042. 17872937

[25] Centers for Diseases Control and Prevention (CDC) . (2022). *The Social‐Ecological Model: A Framework for Prevention*. https://www.cdc.gov/violenceprevention/about/social-ecologicalmodel.html.

[26] Agency for Toxic Substances and Disease Registry , “Models and Frameworks for the Practice of Community Engagement,” in *Principles of Community Engagement* (2nd ed.) (Centers for Disease Control and Prevention and the Agency for Toxic Substances and Disease Registry, 2015), https://www.atsdr.cdc.gov/communityengagement/pce_models.html.

[27] B. C. Lee , C. Bendixsen , A. K. Liebman , and S. S. Gallagher , “Using the Socio‐Ecological Model to Frame Agricultural Safety and Health Interventions,” Journal of agromedicine 22, no. 4 (2017): 298–303, 10.1080/1059924X.2017.1356780. 28762886 PMC11060605

[28] LivingWorks , LivingWorks SafeTALK (LivingWorks Education, 2016b). https://livingworks.com.au/training/livingworks-safetalk/.

[29] LivingWorks , LivingWorks ASIST (LivingWorks Education, 2016a), https://livingworks.com.au/training/livingworks-asist/.

[30] LivingWorks . (2016c). *LivingWorks SafeTALK Training for Trainers (T4T)*. Retrieved May 28 from https://livingworks.com.au/become-a-trainer/livingworks-safetalk-t4t/.

[31] R. Pokharel Poudel , D. Jefferies , S. Perumbil Pathrose , and L. M. Ramjan , “Contextualisation and Evaluation of the Preliminary Effectiveness, Feasibility and Acceptability of the safeTALK Suicide Prevention Programme for Secondary School Students: Protocol for a Multi‐Method Study,” Journal of Clinical Nursing (2025), 10.1111/jocn.70179. 41367059

[32] Prosperity Institute , THE LEGATUM PROSPERITY INDEX™ Advancing the Understanding of National Prosperity (Prosperity Institute, 2023). https://index.prosperity.com/globe/nepal.

[33] K. Malterud , V. D. Siersma , and A. D. Guassora , “Sample Size in Qualitative Interview Studies: Guided by Information Power,” Qualitative Health Research 26, no. 13 (2016): 1753–1760, 10.1177/1049732315617444. 26613970

[34] L. Belone , J. E. Lucero , B. Duran , et al., “Community‐Based Participatory Research Conceptual Model: Community Partner Consultation and Face Validity,” Qualitative Health Research 26, no. 1 (2016): 117–135, 10.1177/1049732314557084. 25361792 PMC4839192

[35] S. E. Langdon , S. L. Golden , E. M. Arnold , et al., “Lessons Learned From a Community‐Based Participatory Research Mental Health Promotion Program for American Indian Youth [Article],” Health Promotion Practice 17, no. 3 (2016): 457–463, 10.1177/1524839916636568. 27009131 PMC9097107

[36] M. L. Phan and T. L. Renshaw , “Guidelines for Implementing and Adapting Evidence‐Based Interventions With Marginalized Youth in Schools,” American Journal of Orthopsychiatry 93, no. 3 (2023): 256–268, 10.1037/ort0000676. 37053428

[37] H. Kyngäs , K. Mikkonen , and M. Kääriäinen , The Application of Content Analysis in Nursing Science Research (Springer International Publishing, 2020). 1st ed, 10.1007/978-3-030-30199-6.

[38] R. Forero , S. Nahidi , J. De Costa , et al., “Application of Four‐Dimension Criteria to Assess Rigour of Qualitative Research in Emergency Medicine,” BMC Health Services Research 18, no. 1 (2018): 120, 10.1186/s12913-018-2915-2. 29454350 PMC5816375

[39] D. B. Goldston , S. D. Molock , L. B. Whitbeck , J. L. Murakami , L. H. Zayas , and G. C. N. Hall , “Cultural Considerations in Adolescent Suicide Prevention and Psychosocial Treatment,” American Psychologist 63, no. 1 (2008): 14–31, 10.1037/0003-066x.63.1.14. 18193978 PMC2662358

[40] T. T. Money and P. J. Batterham , “Sociocultural Factors Associated With Attitudes Toward Suicide in Australia,” Death Studies 45, no. 3 (2021): 219–225, 10.1080/07481187.2019.1626943. 31190630

[41] C. Wang , J. Barlis , K. A. Do , J. Chen , and S. Alami , “Barriers to Mental Health Help Seeking at School for Asian–and Latinx–American Adolescents,” School Mental Health 12, no. 1 (2020): 182–194, 10.1007/s12310-019-09344-y.

[42] L. F. Chan , “Cultures, Contexts, and Learning Opportunities in Suicide Prevention,” Lancet Public Health 9, no. 10 (2024): e716–e717, 10.1016/S2468-2667(24)00216-0. 39265610

[43] R. Thapa , E. van Teijlingen , P. R. Regmi , and V. Heaslip , “Caste Exclusion and Health Discrimination in South Asia: A Systematic Review,” Asia Pacific Journal of Public Health 33, no. 8 (2021): 828–838, 10.1177/10105395211014648. 34024157 PMC8592103

[44] A. Fastenau , P. Chahal , A. Shaheen , and M. Basak , “Risk Factors for Suicide Among South‐East Asian Women: A Public Health Crisis in Need of Gender‐Specific Solutions,” PLOS Mental Health 1, no. 6 (2024): e0000183, 10.1371/journal.pmen.0000183. 41661838 PMC12798182

[45] P. Amatya , S. Ghimire , K. E. Callahan , B. K. Baral , and K. C. Poudel , “Practice and Lived Experience of Menstrual Exiles (Chhaupadi) Among Adolescent Girls in Far‐Western Nepal,” PLoS One 13, no. 12 (2018): e0208260, 10.1371/journal.pone.0208260. 30532183 PMC6287853

[46] S. Thapa and A. R. Aro , “'Menstruation Means Impurity': Multilevel Interventions are Needed to Break the Menstrual Taboo in Nepal,” BMC Women's Health 21, no. 1 (2021): 84, 10.1186/s12905-021-01231-6. 33639917 PMC7971149

[47] A. Atreya , S. Aryal , S. Nepal , and B. Nepal , “Accusations of Witchcraft: A Form of Violence Against Women in Nepal,” Medicine, Science and the Law 61, no. 2 (2021): 147–149, 10.1177/0025802421998222. 33632014

[48] M. Cwik , N. Goklish , K. Masten , et al., “Let Our Apache Heritage and Culture Live on Forever and Teach the Young Ones’: Development of The Elders' Resilience Curriculum, an Upstream Suicide Prevention Approach for American Indian Youth,” American Journal of Community Psychology 64, no. 1–2 (2019): 137–145, 10.1002/ajcp.12351. 31313327

[49] M. S. Gould , F. A. Marrocco , M. Kleinman , et al., “Evaluating Iatrogenic Risk of Youth Suicide Screening Programs: A Randomized Controlled Trial,” Journal of the American Medical Association 293, no. 13 (2005): 1635–1643, 10.1001/jama.293.13.1635. 15811983

[50] J. Jones , “Everyday Nonverbal Communication: A Comparative Study of South and East Asian and the Mid‐Atlantic United States Cultures” (undergraduate theses and Capstone Projects, 2025), https://digitalshowcase.lynchburg.edu/utcp/338/.

[51] Y. Han , N. Aryal , and K. Hwang , “Local Governments' Accountability and Public Trust in Nepal: Does Participation Make a Difference?,” Asia & the Pacific Policy Studies 11, no. 2 (2024): e387, 10.1002/app5.387.

[52] L. Lacey , N. Mishra , P. Mukherjee , et al., “Can Destigmatizing Mental Health Increase Willingness to Seek Help? Experimental Evidence From Nepal,” Journal of Policy Analysis and Management 44, no. 1 (2025): 97–124, 10.1002/pam.22643.

