# Supplementary material for: Contextualisation of the safeTALK™ Suicide Prevention Program: A Descriptive Qualitative Study
Source: Health Expect. 2026 Feb 16;29(1):e70605. doi: 10.1111/hex.70605 (PMC12909602; doi:10.1111/hex.70605)
Supplement: Supplementary file 2 — Supplementary_file_2_Booklet. [file HEX-29-e70605-s002.pdf]

Appendix S2: Booklet

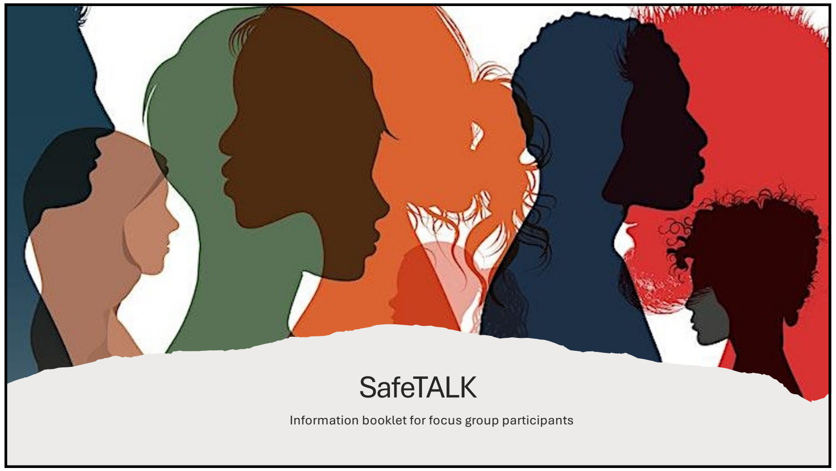

1

About the program

- SafeTALK is a community-wide awareness program that establishes the strongest possible foundations for effective prevention of suicide.
- This program is designed for people aged 15 years or above.

2

About the program

- SafeTALK helps people to recognize ‘invitations’ when a person is considering suicide.
- The person considering suicide initiates communications that invite assistance, the invitation may be unique to everyone. E.g.: some people may communicate, others may not verbalise, and they may just show the gesture.
- Helpers must then choose how to respond to these invitations—whether to connect with suicide or miss, dismiss, or avoid such safety-seeking communications.
- Helpers can then work with the person to make one or more safe connections with others who can provide a suicide intervention, with a particular initial focus on suicide first aid.

3

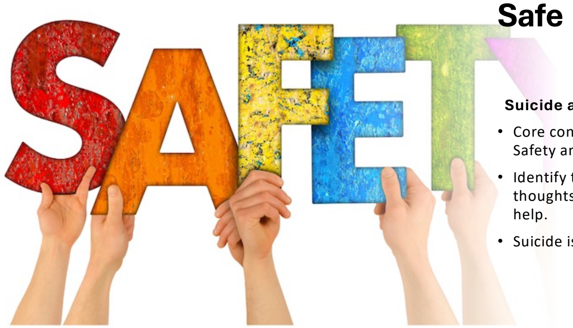

Safe

Suicide alertness for everyone

- Core concepts in safeTALK are Safety and Connectedness.
- Identify the person with suicidal thoughts and connect them with help.
- Suicide is everyone’s business.

4

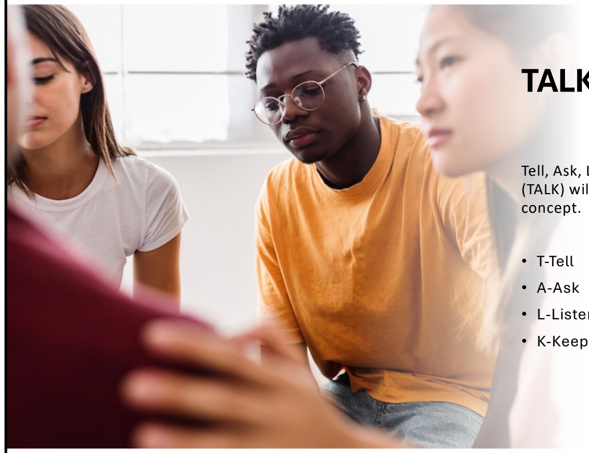

TALK

Tell, Ask, Listen and KeepSafe (TALK) will inform the connecting concept.

- T-Tell
- A-Ask
- L-Listen
- K-Keep Safe

5

Tell

- There may be different ways to tell by different people
  - Actions: Careless/moody/withdrawing/misuse of drugs or alcohol
  - Talk: alone, no purpose in life, burden, want to escape
  - Feeling: Desperate, hopeless, numb, ashamed
  - Life Situations: abuse, rejection, losses, suicidal experience

6

Questions based on Tell to reflect on

1. What are the common actions Nepalese adolescents may use to express suicide?

____

2. What are the common words that may be used by Nepalese adolescents to talk about their suicidal thoughts?

____

3. Are there any different or additional feelings Nepalese adolescents may express?

____

4. What life situations may be the contributors of adolescent suicide in Nepal?

____

7

Ask

- Asking a direct question about suicidal thoughts
  - When a person shows invitations ask directly **"Are you thinking about suicide?"**

Questions to reflect on

1. What is your opinion about asking direct questions about suicidal thoughts?

____

2. What are some culturally appropriate and effective ways to ask about suicidal thoughts?

____

8

Listen

- **Let's talk about this…… I am listening……….that is important**

Questions to reflect on

1. What is the most culturally appropriate way to listen actively in Nepal?

____

2. How would you tell someone that you are open to listening to them?

____

9

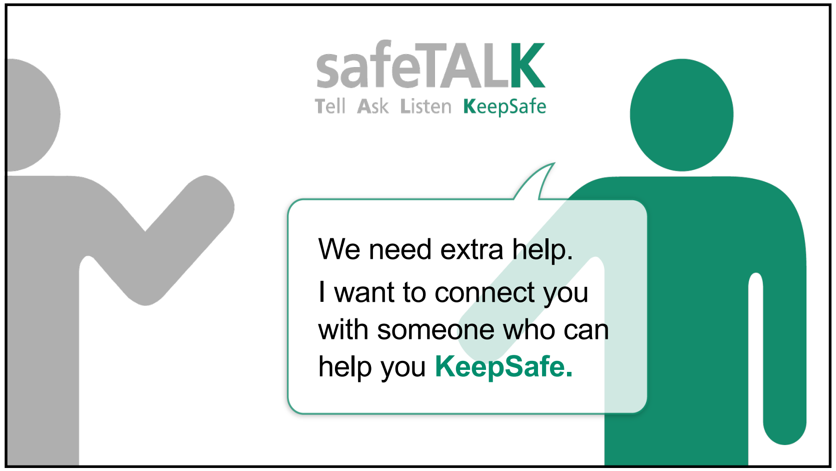

10

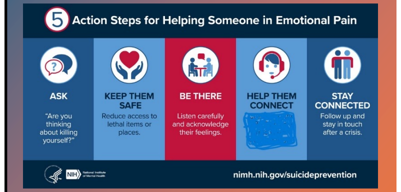

KeepSafe

A question to reflect on

1. What are appropriate resources that we can use to keep people safe from Suicide in Nepal?

____

____

____

11
