# Supplementary material for: Contextualisation of the safeTALK™ Suicide Prevention Program: A Descriptive Qualitative Study
Source: Health Expect. 2026 Feb 16;29(1):e70605. doi: 10.1111/hex.70605 (PMC12909602; doi:10.1111/hex.70605)
Supplement: Supplementary file 3 — Supplementary_file_3_Focus_group_questions. [file HEX-29-e70605-s004.pdf]

Appendix S3: Focus group discussion plan

Schedule

	Activities	Time in minutes
1.	Welcome	2
2.	Objective	1
3.	Ground Rules	2
4.	Participant Introductions	2
5.	Assessment of the need for the program (Discuss Question 1)	5
6.	Presentation of the program	20
7.	Discuss other questions	30-60
8.	Conclude the session	10

Welcome and self-Introduction

If you feel distressed due to the contents of the discussion, you are free to leave the session and contact the support person available there.

I have a few questions to discuss, but the session will not be limited to this content. You are free to add more relevant points if you have any.

Objective of the session

To discuss the SafeTALK™ suicide prevention program and its cultural appropriateness in Nepal.

Ground rules

1. Please maintain the confidentiality of other members in this group, do not disclose personal information of another member from today's discussion outside this group.
2. Please keep your phone on silent mode during the discussion.
3. Please mute your microphone while others are speaking.
4. This session is being recorded, therefore please avoid unnecessary noises.
5. There will not be any right or wrong opinions, so you are free to give your opinion. Everyone's opinion is equally powerful in this discussion.
6. We will not discuss our personal story here.

Focus group discussion guide

For adolescents

1. Do you think that an adolescent suicide prevention program is needed in Nepal? Please explain your reasons.
   A presentation will be conducted after discussing question 1. A video without a co-trainer will be briefly presented to orientate participants with core concepts of SafeTALK™. For the presentation in Nepali, the researcher will prepare notes in Nepali Language before running the focus groups.

2. Is the SafeTALK™ program appropriate for young people your age at schools in Nepal? Please explain your reasons.

3. Could anything be added or removed from the existing program? Please explain your thoughts further.
4. Why do you think it is important to remove or add those elements?
5. How do family structure and social support affect suicide in Nepal?
   Could you please tell me about your opinion on each component of SafeTALK™?

1. Suicide alertness for everyone (Safe)	
2. Tell	
3. Ask	
4. Listen	
5. Keep safe	
Other things if you want to add	

For Adults (Parents, teachers, healthcare providers, and policymakers)

1. What are your thoughts on the need for a suicide prevention program in secondary schools in Nepal?
   A presentation will be conducted after discussing question 1. A video without a co-trainer will be briefly presented to orientate participants to the core concepts of SafeTALK™. For the presentation in Nepali, the researcher will prepare notes in Nepali Language before running focus groups. Before presenting the program to policymakers or healthcare providers, discuss the identified gap in the review. For the presentation in Nepali, the researcher will prepare notes in Nepali language before running the focus groups.
2. Is the SafeTALK™ program appropriate for the Nepalese context? Why or why not?
3. What modifications are required to the existing program? Why are these necessary?
4. What needs to be added or removed from the existing program for it to be more culturally appropriate?
