# Supplementary material for: Contextualisation of the safeTALK™ Suicide Prevention Program: A Descriptive Qualitative Study
Source: Health Expect. 2026 Feb 16;29(1):e70605. doi: 10.1111/hex.70605 (PMC12909602; doi:10.1111/hex.70605)
Supplement: Supplementary file 4 — Supplementary_file_4_Approved_modifications. [file HEX-29-e70605-s003.pdf]

Appendix S4: Final approved modifications to SafeTALK™

Slide number in SafeTALK™ training	Proposed changes and approvals
5 -7 (videos) and all other videos	**Proposed:** Nepali subtitles in all videos that provide the main suicide prevention messages.<br>**Approved:** Nepali subtitles added to the videos delivering the main messages.
	**Proposed:** Nepali people and contextually appropriate settings would be more effective.<br>**Approved:** Two animations were developed to preface the training to capture a realistic context for the community and to engage the students.
16	**Proposed:** Tell: not eating, locking oneself in the room.<br>**Approved:** These additional 'Tell' signs to be explained during program delivery.
17	**Proposed:** Additional points to discuss in Tell, such as: Bullying (cyberbullying) or any kind of torture, writing suicidal posts on social media, gender discrimination by family members and unexpected low rank in the class.<br>**Approved:** Explain these points in Tell during delivery.
18	**Proposed:** Discuss the following in Life Situations.<br>1. Peculiar cultural practice in some of the communities in Nepal; being accused of witchcraft, dowry systems, Chhaupadi.<br>2. Social structure; hierarchical caste system and untouchability on the basis of caste.<br>3. Unnecessary gossip and involvement of neighbours and relatives in family matters puts adolescents under extreme pressure to perform well in all aspects of life.<br>4. Poverty and food insecurity, domestic violence especially towards females by their in-laws.<br>5. Early marriage, hidden romantic relationship with unmarried pregnancy which is not accepted by families.<br>6. Peer pressure to engage in certain behaviours, for example drug and alcohol use.<br>**Approved:** Discussion of the above cultural practices during delivery of Life Situations.
21	**Proposed:** The word INVITATIONS needs to be contextualised during translation into the Nepali language. For example, participants explained that it can be seen as an invitation for suicide not for prevention.<br>**Approved:** The word Invitations was retained however there was an explanation that Invitations save people's lives as they show symptoms or tell us things that suggest suicide.
21	**Proposed:** Discuss the following in ASK<br>In certain communities especially Muslim and Madhesi communities, females may not feel comfortable being asked questions by males, so this needs to be explained during the training.<br>**Approved:** Discussion of the above cultural consideration.
29	**Proposed:** Discuss the following:<br>Techniques for active listening may differ from culture to culture within Nepal. Some people may prefer eye contact and sustain it, while others may feel threatened.<br>**Approved:** Discussion of the above cultural consideration.
30	**Proposed:** Replace the KeepSafe contacts list with Nepal specific contacts.<br>**Approved:** Replacement with a separate contact list of helplines for Nepal.
