# Supplementary material for: Contextualisation of the safeTALK™ Suicide Prevention Program: A Descriptive Qualitative Study
Source: Health Expect. 2026 Feb 16;29(1):e70605. doi: 10.1111/hex.70605 (PMC12909602; doi:10.1111/hex.70605)
Supplement: Supplementary file 5 — Supplementary_file_5_Animation_videos. [file HEX-29-e70605-s001.pdf]

Appendix S5: Short animation videos

Supplementary Video 1: Gita's story
YouTube link: https://youtu.be/RwXJ1P-KAdU?feature=shared
Description: Gita, a 14-year-old girl in Nepal, experiencing societal pressure

Supplementary Video 2: Dhan's story
YouTube link: https://youtu.be/yp3XsYmyJOU?feature=shared
Description: Dhan, a 14-year-old boy in Nepal, experiencing academic pressure
